# Isobaric Tagging-Based Quantification for Proteomic Analysis: A Comparative Study of Spared and Affected Muscles from *mdx* Mice at the Early Phase of Dystrophy

**DOI:** 10.1371/journal.pone.0065831

**Published:** 2013-06-18

**Authors:** Cintia Yuri Matsumura, Bruno Menezes de Oliveira, Madeleine Durbeej, Maria Julia Marques

**Affiliations:** 1 Departamento de Biologia Estrutural e Funcional, Instituto de Biologia, Universidade de Campinas (UNICAMP), Campinas, São Paulo, Brazil; 2 Muscle Biology Unit, Department of Experimental Medical Science, University of Lund, Lund, Sweden; University of Edinburgh, United Kingdom

## Abstract

Duchenne muscular dystrophy (DMD) is the most common childhood myopathy, characterized by muscle loss and cardiorespiratory failure. While the genetic basis of DMD is well established, secondary mechanisms associated with dystrophic pathophysiology are not fully clarified yet. In order to obtain new insights into the molecular mechanisms of muscle dystrophy during earlier stages of the disease, we performed a comparative proteomic profile of the spared extraocular muscles (EOM) *vs.* affected diaphragm from the *mdx* mice, using a label based shotgun proteomic approach. Out of the 857 identified proteins, 42 to 62 proteins had differential abundance of peptide ions. The calcium-handling proteins sarcalumenin and calsequestrin-1 were increased in control EOM compared with control DIA, reinforcing the view that constitutional properties of EOM are important for their protection against myonecrosis. The finding that galectin-1 (muscle regeneration), annexin A1 (anti-inflammatory) and HSP 47 (fibrosis) were increased in dystrophic diaphragm provides novel insights into the mechanisms through which *mdx* affected muscles are able to counteract dystrophy, during the early stage of the disease. Overall, the shotgun technique proved to be suitable to perform quantitative comparisons between distinct dystrophic muscles and allowed the suggestion of new potential biomarkers and drug targets for dystrophinopaties.

## Introduction

Duchenne muscular dystrophy (DMD) is the most common and devastating of the human muscular dystrophies. It is characterized by progressive muscle weakness and death from cardiorespiratory compromise around the second or third decade of life [1]–[3]. In DMD and in the *mdx* mice model of DMD [4], [5] the genetic abnormality is in the X chromosome, in which the nucleotide sequence responsible for the expression of the protein dystrophin is mutated. In the absence of dystrophin, instability of sarcolemma leads to progressive myonecrosis, followed by intense inflammation and fibrosis [1], [6].

Numerous proteomics analysis of dystrophic muscles in *mdx* and in DMD have been performed with the aim to unravel the molecular pathogenesis of muscular dystrophy [7]–[13]. Previous proteomic studies included the use of the differential gel electrophoresis (DIGE), which provided important data related to the nature of the dystrophic proteome and showed a great number of proteins that were differentially expressed in distinct dystrophic muscles and ages [7], [10]–[15].

The multidimensional protein identification technology (MudPIT) method is a gel free alternative [16]–[18] to conventional gel-based methods, which has revolutionized the proteomic field. Basically, complex proteins mixtures are digested to peptides, fractionated according to its different chemical properties and subsequently analyzed by tandem mass spectrometry (MS/MS) resulting in protein identification. By using MudPIT it is possible to overcome several drawbacks associated with two-dimensional gel electrophoresis (2D-PAGE), especially under-representation of extreme acid/basic proteins [19] and the poor sensitivity for lowly expressed proteins. Moreover the MudPIT method simplifies sample handling, avoids sample loss in gel matrix and increases throughput and data acquisition [20], [21].

Protein quantification is fundamental for any comparative proteomic study of biological systems. In a proteomic analysis, the number of extracted proteins in a sample is higher than the number of identified proteins, which in turn is higher than the total number of quantified proteins [22]. In MS-based proteomics, two basic possibilities of quantification exist: (*i*) a relative quantification of proteins in compared samples (e.g. control *vs.* disease state) or (*ii*) an absolute quantification [23]. Isobaric mass tagging reagents, as tandem mass tags (TMT), allow multiple and independent measures of protein abundance in the same experiment, enabling statistical estimates of protein quantification and comparisons between different samples [24].

In order to obtain new insights into the molecular mechanisms of muscle dystrophy, we here used a label based shotgun proteomic approach, combining TMT labels and MudPIT method to analyze the diaphragm of 2-month-old *mdx* mice. We considered the age of 2 months as an early phase of dystrophy, similar to other proteomic studies [12], given the worsening of disease overtime. Signs of necrosis have been reported to occur at about 5 weeks of age in the *mdx* diaphragm [25]. At 2 months of age, diaphragm shows active muscle damage, regeneration and inflammation. However, significant histopathological signs of the disease, such as extensive fibrosis, are not seen at this age, suggesting an ability to compensate for muscle degeneration with cycles of muscle regeneration, as reported for limb muscles [26], [27]. Fibrosis will be present later in *mdx* diaphragm, increasing progressively from 6 months to 1 year of age and onwards, a timepoint when the muscle mostly resembles DMD myopathy [13], [26], [28]. In our proteomic analysis we have also searched for compensatory mechanisms involved in dystrophic muscle protection against myonecrosis, by comparing the proteomic profiles from dystrophic *mdx* diaphragm against the one from *mdx* extraocular muscles, which do not show muscle degeneration [29].

## Materials and Methods

### Animals

Males and females, 2-month-old *mdx* (C57BL/10ScSn-mdx/J) mice (n = 15) and age-match wild-type mice (C57BL/10SnJ, n = 15) were obtained from Jackson Laboratory and maintained in the animal facilities of Biomedical Center (Lund) according to the animal care guidelines. All mouse experimentation was approved by the Malmö/Lund (Sweden) ethical committee for animal research (permit numbers M62-09 and M122-10).

### TMT labelling of mouse muscle samples

#### Protein extraction and preparing of the samples

**Figure 1:**
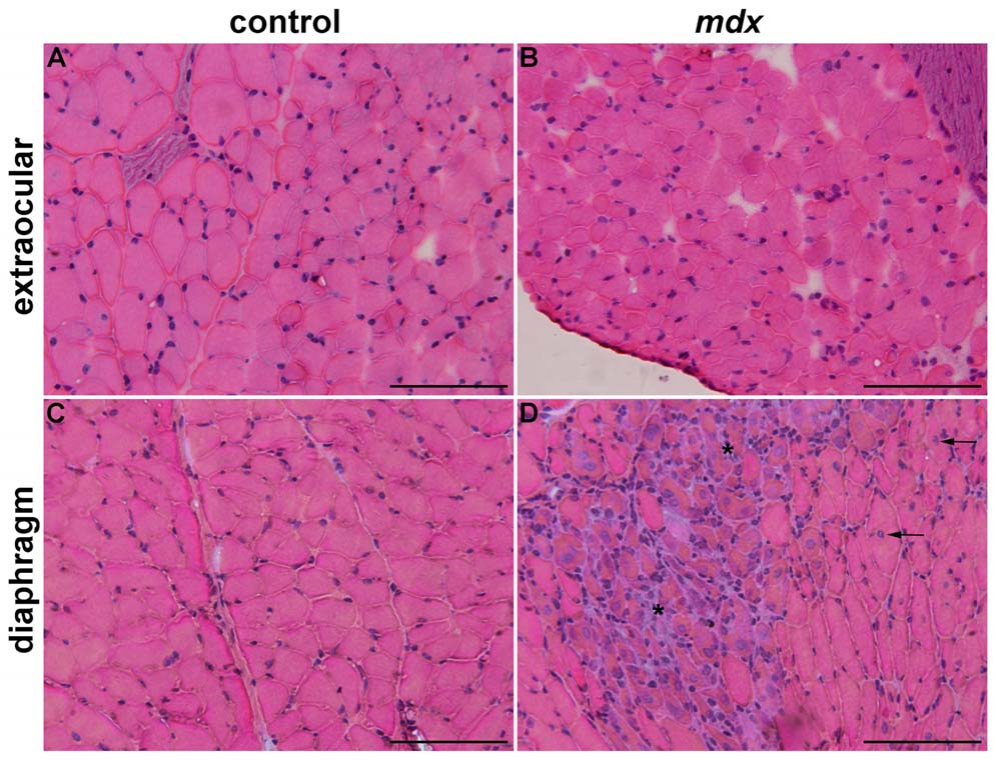
Histological differences between extraocular and diaphragm muscles. Extraocular (A, B) and diaphragm (C, D) muscles of control (A, C) and *mdx* (B, D) mice. In controls (A, C), muscle fibers show peripheral nuclei. In the spared dystrophic EOM (B), fibers with peripheral nuclei indicate lack of muscle degeneration-regeneration. In the *mdx* DIA (D), fibers with central nuclei (arrows in D; regenerated fibers), and areas containing muscle fibers in regeneration surrounded by inflammatory cells (asterisk). Scale bar: 100 µm.

Mice were sacrificed by cervical dislocation and the diaphragm (DIA; Figure 1) muscle and extraocular muscles (EOM; Figure 1) were dissected out, frozen in liquid nitrogen and reduced to powder using a mortar. Three different pools for each group (*mdx* mice and wild-type mice) were made, each composed of five animals. The muscles were lysed in assay lysis buffer (10 mM NaHCO3, 5% SDS) containing freshly added protease and phosphatase inhibitors (Roche - Indianapolis, IN, USA). The samples were centrifuged for 10 min at 15,682 g, and the soluble fraction was removed. The protein concentration was determined using BCA Protein Assay Kit (Pierce).

The samples were processed according to the instructions of the TMT isobaric Mass Tagging Kits and Reagents. In brief, 100 µg of protein per condition were mixed in six volumes of pre-chilled (−20°C) acetone and precipitated overnight. After centrifugation at 8,000 g for 10 minutes at 4°C, the pellet was dried. For protein digestion, 5 µl of 2% SDS, 45 µl of 200 mM TEAB were added to the sample and the final volume was adjusted to 100 µl with ultrapure water. Five microliter of 200 mM TCEP were added to the sample and incubated at 55°C for 1 hour. Then, 5 µl of the 375 mM iodoacetamide (with TEAB) were added and incubated for 30 min protected from light. To digest proteins, 2.5 µg of trypsin were added and kept overnight at 30–37°C. For protein labeling, 41 µl of the TMT Label Reagent were added to each sample and incubated for 1 h at room temperature. Eight microliter of 5% hydroxylamine were added and incubated for 15 min. Our labeling design allowed a label swap, in order to avoid possible bias due to technical errors (Table 1).

**Table 1:** Experimental setup for 2 muscles (EOM - extraocular and DIA - diaphragm) under 2 conditions (control and *mdx*) and with 3 biological replicates (Pool 1, 2 and 3).

	Groups	TMT label
**Pool 1** (n = 5)	EOM control	127
	DIA control	128
	EOM *mdx*	129
	DIA *mdx*	130
	Internal standard	126
**Pool 2** (n = 5)	EOM control	130
	DIA control	129
	EOM *mdx*	128
	DIA *mdx*	127
	Internal standard	126
**Pool 3** (n = 5)	EOM control	129
	DIA control	130
	EOM *mdx*	127
	DIA *mdx*	128
	Internal standard	126

The internal standard is a mixture of all samples.

#### SCX fractionation of the pooled TMT labelled samples

The pooled TMT-labelled samples were fractionated by strong cation-exchange (SCX, Applied Biosystems) using 500 µl of buffer A with 30, 60, 90, 120, 240, 300, 420, 500 mM KCl, respectively and collect as fractions 1–8, respectively. The fractions were cleaned on Ultra Microspin C18 columns (The Nest Group, Southboro, MA, USA), dried and resuspended in 30 µl 0.1% formic acid.

#### LC-MS/MS Analysis on LTQ-OrbitrapXL

The fractions were analyzed on an LTQ-OrbitrapXL (Thermo Fisher Scientific) interfaced with an in-house constructed nano-LC column. Two-microliter sample injections were made with an HTC-PAL autosampler (CTC Analytics AG) connected to an Agilent 1200 binary pump (Agilent Technologies). The peptides were trapped on a pre-column (40×0.075 mm i.d.) and separated on a reversed phase column, 200×0.05 mm. Both columns were packed in-house with 3 µm Reprosil-Pur C18-AQ particles. The flow through the analytical column was reduced by a split to approximately 100 nl/min and the gradient was as followed; 0–6 min 0.1% formic acid, 6–76 min 7–35% acetonitrile, 0.1% formic acid, 76–79 min 40–80% acetonitrile 0.1% formic acid.

LTQ-OrbitrapXL settings were: spray voltage 1.4 kV, 1 microscan for MS1 scans at 60 000 resolutions (m/z 400), full MS mass range m/z 400–2000. The LTQ-Orbitrap XL was operated in a data-dependent mode with one MS1 FTMS scan of precursor ions followed by CID (collision induced dissociation) and HCD (high energy collision dissociation), MS2 scans of the three most abundant doubly, triply and quadruply protonated ions in each FTMS scan. The settings for the MS2 were as follows: 1 microscans for HCD-MS2 at 7500 resolution (at m/z 400), mass range m/z 100–2000 with a collision energy of 50%, 1 microscans for CID-MS2 with a collision energy of 30%. Dynamic exclusion of a precursor selected for MS2 was used for 120 s after one repeat, enabling most of the co-eluting precursors to be selected for MS2.

#### Database Search and TMT Quantification

MS raw data files from all 8 SCX fractions per one TMT set and 3 MS runs were merged for relative quantification and identification using Proteome Discoverer version 1.3 (Thermo Fisher Scientific), respectively. Database search was performed by Mascot search engine using the following critera: *Mus musculus* in Swissprot protein database from April 2012, MS peptide tolerance as 10 ppm, MS/MS tolerance as 0.5 Da, trypsin digestion allowing 1 missed cleavages with variable modifications; methionine oxidation, cysteine methylthiol, and fixed modifications; N-terminal TMT6-plex label, lysine TMT6-plex label. The detected protein threshold in the software was set to a confidence using the FDR 1% method and identified proteins were grouped by sharing the same sequences to minimize redundancy.

For quantification, the ratios of TMT reporter ion intensities in MS/MS spectra (m/z 126.12, 127.13, 128.13, 129.14, 130.14) from raw data sets were used to calculate fold changes between samples via the relative ratio to the reference pool. Only peptides unique for a given protein were considered for relative quantitation, excluding those common to other isoforms or proteins of the same family. Only peptides with a score >10 and below the Mascot significance threshold filter of *p* = 0.05 were included. Single peptide identifications required a score equal to or above the Mascot identity threshold. Normalisation on protein median was used. The median of peptides was used for protein ratio and the resulting ratios were then exported into Excel for manual data interpretation.

Statistical analysis was performed by Student's t-test, with p values ≤0.05, with protein ratios smaller than −1.25 or greater than 1.25 and a coefficient of variation of less than 20% considered significantly different. For correction of false-positive values the FDR (false discovery rate) with estimated q-values was used [30].

### Western blot analysis

Verification of the proteomic findings was carried out by comparative Western blot analysis. The levels of galectin-1 (Gal), annexin A5 (ANXA 5), β-dystroglycan (β-DG), calmodulin I (CaM) and calsequestrin-1 (CSQ) were quantified in DIA and EO muscles of control (n = 6) and *mdx* (n = 6) mice. The method was previously described [28], [31]. Muscles were lysed in assay lysis buffer containing freshly added protease and phosphatase inhibitors (1% Triton, 10 mM sodium pyrophosphate, 100 mM NaF, 10 µg/ml aprotinin, 1 mM PMSF, and 0.25 mM Na_3_VO_4_). The samples were centrifuged for 20 min at 12,581 g, and the soluble fraction was resuspended in 50 µl Laemmli loading buffer (2% SDS, 20% glycerol, 0.04 mg/ml bromophenol blue, 0.12 M Tris-HCl, pH 6.8, and 0.28 M ß-mercaptoethanol). An amount of 60 µg of total protein homogenate was loaded onto 8%–15% SDS-polyacrylamide gels. Proteins were transferred from the gels to a nitrocellulose membrane using a submersion electrotransfer apparatus (Bio-Rad Laboratories, Hercules, California, USA). Membranes were blocked for 2 h at room temperature with 5% skim milk/Tris-HCl buffered saline-Tween buffer (TBST; 10 mM Tris-HCl, pH 8, 150 mM NaCl, and 0.05% Tween 20). The membranes were incubated with the primary antibodies overnight at 4°C, washed in TBST, incubated with the peroxidase-conjugated secondary antibodies for 2 h at room temperature, and developed using the SuperSignal West Pico Chemiluminescent Substrate kit (Pierce Biotechnology, Rockford, Illinois, USA). To control for protein loading, Western blot transfer and nonspecific changes in protein levels, the blots were stripped and re-probed for glyceraldehyde-3-phosphate dehydrogenase (GAPDH). The signal from western blotting bands was captured (G:Box iChemi camera; Syngene, Cambridge, UK) and quantified using the software Gene Tools Version 4.01, Syngene, Cambridge, UK.

The following primary antibodies were used: annexin A5, galectin-1, calmodulin I and GAPDH (Santa Cruz Biotechnology, Santa Cruz, California, USA); β-dystroglycan (Novocastra Laboratories Ltd., Benton Lane, Newcastle Upon Tyne, UK); and calsequestrin-1 (Affinity Bioreagents, Golden, Colorado, USA). The corresponding secondary antibody was peroxidase-labeled affinity-purified mouse or rabbit IgG antibody (H+L) (KPL, Gaithersburg, Maryland, USA).

## Results

### Shotgun proteomic analysis of DIA and EOM

By using the shotgun with MudPIT and TMT methodology, a total of 857 proteins were identified (DIA plus EOM). From this, about 48% (415 out of 857; Table S1) were common to the muscles studied, i.e., they were detected in both EOM and DIA, from both conditions (control and *mdx*) and in the three biological replicates. The criteria to identify proteins as being significantly changed between samples were proteins ratios with p-value ≤0.05 (Student's t-test), q-value ≤0.025 and protein ratios smaller than −1.25 or greater than 1.25. About 10% (42 out of 415; Table 2) of the proteins showed an altered expression pattern in the dystrophic DIA compared with the control DIA. We did not observe any proteins that had differential abundance of peptide ions in the *mdx* EOM (compared to control EOM), according to our established criteria. Overall, the proteins identified could be grouped into several biological processes such as immune system processes, energy and metabolism, sarcomeric and cytoskeletal proteins.

**Table 2:** The identified proteins that exhibit change in abundance in *mdx* diaphragm in comparison to control diaphragm.

Accession	Description	MW [kDa]	ΣCoverage	Σ# Peptides	Fold Change
Q5SX39	Myosin-4	222.7	57.9	133	−1.49
P13707	Glycerol-3-phosphate dehydrogenase [NAD+], cytoplasmic	37.5	15.5	5	−1.45
Q8BW75	Amine oxidase [flavin-containing] B	58.5	2.7	2	−1.42
Q80XN0	D-beta-hydroxybutyrate dehydrogenase, mitochondrial	38.3	8.8	3	−1.35
P19157	Glutathione S-transferase P 1	23.6	12.9	2	−1.33
P10649	Glutathione S-transferase Mu 1	26.0	28.9	6	−1.32
P28665	Murinoglobulin-1	165.2	3.1	5	−1.30
P68134	Actin, alpha skeletal muscle	42.0	58.1	22	−1.30
P09542	Myosin light chain 3	22.4	45.1	8	−1.30
Q924D0	Reticulon-4-interacting protein 1, mitochondrial	43.3	3.3	1	−1.30
P07310	Creatine kinase M-type	43.0	55.4	21	−1.29
Q9D0F9	Phosphoglucomutase-1	61.5	10.0	5	−1.29
O09165	Calsequestrin-1	45.6	23.8	10	−1.29
Q91V92	ATP-citrate synthase	119.7	3.2	3	−1.29
Q9CQ62	2,4-dienoyl-CoA reductase, mitochondrial	36.2	27.2	8	−1.28
P20801	Troponin C, skeletal muscle	18.1	37.5	5	−1.27
P05064	Fructose-bisphosphate aldolase A	39.3	44.5	18	−1.26
O70250	Phosphoglycerate mutase 2	28.8	24.5	8	−1.26
P14152	Malate dehydrogenase, cytoplasmic	36.5	28.1	7	−1.25
Q9CQN1	Heat shock protein 75 kDa, mitochondrial	80.2	4.4	2	1.26
P19253	60S ribosomal protein L13a	23.4	3.9	1	1.28
P20029	78 kDa glucose-regulated protein	72.4	10.2	5	1.28
P35980	60S ribosomal protein L18	21.6	6.9	1	1.31
P09103	Protein disulfide-isomerase	57.1	12.8	7	1.32
Q8VDD5	Myosin-9	226.2	6.3	11	1.33
P47915	60S ribosomal protein L29	17.6	5.0	1	1.41
Q9Z1N5	Spliceosome RNA helicase Bat1	49.0	1.4	1	1.41
Q9CZX8	40S ribosomal protein S19	16.1	18.6	3	1.42
Q9D1R9	60S ribosomal protein L34	13.3	6.0	1	1.45
Q91VR5	ATP-dependent RNA helicase DDX1	82.4	1.8	1	1.47
P47955	60S acidic ribosomal protein P1	11.5	14.0	1	1.54
P14148	60S ribosomal protein L7	31.4	12.2	3	1.59
Q8CGP6	Histone H2A type 1-H	13.9	27.3	3	1.61
P19324	Serpin H1	46.6	10.8	3	1.62
P48036	Annexin A5	35.7	23.2	8	1.65
P17742	Peptidyl-prolyl cis-trans isomerase A	18.0	35.4	6	1.71
Q8CI43	Myosin light chain 6B	22.7	34.3	7	1.75
P20152	Vimentin	53.7	43.4	18	1.77
P51881	ADP/ATP translocase 2	32.9	38.9	12	1.95
P16045	Galectin-1	14.9	5.9	1	2.02
Q61171	Peroxiredoxin-2	21.8	14.7	2	2.03
P10107	Annexin A1	38.7	10.4	3	2.19

### Comparative proteomic profiling: mdx DIA versus control DIA

By comparing dystrophic DIA with control DIA, we found that most of the proteins detected were present in the sarcoplasm or in the cytoskeleton. Mitochondrion was the organelle that displayed the highest percentage of proteins with altered levels, followed by the nucleus and the sarcoplasmic reticulum (Table 3). The majority of the identified proteins belonged to the class of metabolic proteins or to the class of immune system processes. All 8 protein metabolism-related ribosomal proteins had increased expression in the *mdx* DIA in comparison to control DIA. Regarding the immune system process, 2 proteins involved in responses to toxins (glutathione S-transferase P1 and glutathione S-transferase Mu1) were decreased in the *mdx* DIA compared to control DIA while 7 proteins were increased in the *mdx* DIA compared with control DIA, including proteins involved in responses to stress (heat shock protein 75 kDa, mitochondrial and 78 kDa glucose-regulated protein), induction of apoptosis (galectin-1), oxidative (ROS) processes (peroxiredoxin-2) and finally serine-type endopeptidase inhibitors (serpin H1 and murinoglobulin-1). Proteins involved in cellular respiration were also decreased in the *mdx* DIA, with 3 engaged in glycolysis (phosphoglucomutase-1, fructose-bisphosphate aldolase A and phosphoglucerate mutase 2), 2 in tricarboxylic acid cycle (ATP-citrate synthase and malate dehydrogenase) and 1 from the respiratory electron transport chain (amine oxidase [flavin-containing] B).

**Table 3:** Localization of shotgun identified proteins with altered expression in the comparisons: *mdx* diaphragm (DIA)×control (ct) DIA; ct DIA×ct extraocular (EO); *mdx* DIA×*mdx* EO.

	*mdx* DIA×ct DIA	ct DIA×ct EO	*mdx* DIA×*mdx* EO
**Sarcoplasm**	20.9%	19.4%	30.0%
**Cytoskeleton**	30.2%	21.0%	14.3%
**Extracellular Matrix**	7.0%	3.2%	10.0%
**Mitochondrion**	18.6%	29.0%	17.1%
**Nucleus**	14.0%	9.7%	4.3%
**Sarcolemma**	2.3%	8.1%	15.7%
**Sarcoplasmic reticulum**	4.7%	6.5%	8.6%
**Peroxisome**	2.3%	3.2%	0.0%

### Comparative proteomic profiling: DIA versus EOM

About 13% (54 out of 415; Table 4) of the proteins showed differential abundance of peptide ions when comparing control DIA with control EOM and 15% (62 out of 415; Table 5) increased or decreased in the *mdx* DIA compared with the *mdx* EOM. By performing a further double comparison (i.e., control DIA×control EOM with *mdx* DIA×*mdx* EOM), 21 proteins were found in common, and may represent constitutive proteins related to embryological, morphological or functional differences between DIA and EOM muscles, rather than related to the pathogenesis of dystrophy *per se*. Among these 21 proteins, only annexin A1 showed a distinct pattern of change (increased or decreased) depending on the comparison (39% decreased in control DIA *vs.* control EOM and 51% increased in *mdx* DIA *vs. mdx* EOM). The remaining 20 proteins, most [11] were decreased in DIA *vs.* EOM (collagen alpha-1 and alpha-2; myosin 3, 4 and 11; SERCA 1; calsequestrin 1; sarcalumenin; aspartate aminotransferase; tropomyosin alpha -3 and mitochondrial 2-oxoglutarate/malate carrier protein), with a few [9] proteins increased in DIA×EOM (voltage-dependent anion selective channel protein 1; myosin 1, regulatory light chain 2 and light chain 3; uncharacterized protein C1orf93 homolog; isocitrate dehydrogenase (NADP) mitochondrial; L-lactate dehydrogenase B chain; C-X-C chemokine receptor type 1, and SERCA 2).

**Table 4:** The identified proteins that exhibit change in abundance in control diaphragm in comparison to control extraocular muscle.

Accession	Description	MW [kDa]	ΣCoverage	Σ# Peptides	Fold Change
P08121	Collagen alpha-1(III) chain	138.9	0.8	1	−4.12
P13541	Myosin-3	223.7	31.8	66	−3.42
O08638	Myosin-11	226.9	4.6	8	−2.44
Q8CI43	Myosin light chain 6B	22.7	34.3	7	−2.31
Q01149	Collagen alpha-2(I) chain	129.5	4.7	6	−2.28
Q7TQ48	Sarcalumenin	99.1	27.8	18	−2.18
Q9CR62	Mitochondrial 2-oxoglutarate/malate carrier protein	34.1	12.4	4	−2.04
Q5SX39	Myosin-4	222.7	57.9	133	−1.87
Q8R429	Sarcoplasmic/endoplasmic reticulum calcium ATPase 1	109.4	31.7	31	−1.76
Q6PIE5	Sodium/potassium-transporting ATPase subunit alpha-2	112.1	10.9	11	−1.63
P05202	Aspartate aminotransferase, mitochondrial	47.4	22.8	10	−1.51
P10922	Histone H1.0	20.8	20.6	4	−1.43
P10107	Annexin A1	38.7	10.4	3	−1.39
Q8CGP6	Histone H2A type 1-H	13.9	27.3	3	−1.36
P21107	Tropomyosin alpha-3 chain	32.8	49.3	21	−1.35
O09165	Calsequestrin-1	45.6	23.8	10	−1.33
Q00896	Alpha-1-antitrypsin 1–3	45.8	22.3	11	−1.28
Q6PB66	Leucine-rich PPR motif-containing protein, mitochondrial	156.5	3.1	5	−1.28
Q60714	Long-chain fatty acid transport protein 1	77.9	19.9	12	−1.25
O88346	Troponin T, slow skeletal muscle	31.3	7.6	2	1.25
P97447	Four and a half LIM domains protein 1	31.9	16.1	5	1.26
Q8BMS1	Trifunctional enzyme subunit alpha, mitochondrial	51.4	29.5	14	1.27
P50544	Very long-chain specific acyl-CoA dehydrogenase, mitochondrial	70.8	13.0	7	1.27
P51174	Long-chain specific acyl-CoA dehydrogenase, mitochondrial	47.9	25.8	12	1.28
Q07417	Short-chain specific acyl-CoA dehydrogenase, mitochondrial	44.9	10.2	3	1.28
Q99JY0	Trifunctional enzyme subunit beta, mitochondrial	82.6	25.7	19	1.32
P10649	Glutathione S-transferase Mu 1	25.9	28.9	6	1.32
P58771	Tropomyosin alpha-1 chain	32.7	72.2	34	1.34
Q9CQ62	2,4-dienoyl-CoA reductase, mitochondrial	36.2	27.2	8	1.35
Q99LX0	Protein DJ-1	20.0	8.0	2	1.35
P15626	Glutathione S-transferase Mu 2	25.7	16.1	4	1.35
Q60932	Voltage-dependent anion-selective channel protein 1	32.3	41.6	10	1.37
Q9DB60	Uncharacterized protein C1orf93 homolog	21.7	4.0	1	1.39
Q91WC3	Long-chain-fatty-acid–CoA ligase 6	78.0	2.7	2	1.39
P07310	Creatine kinase M-type	43.0	55.4	21	1.40
Q924X2	Carnitine O-palmitoyltransferase 1, muscle isoform	88.2	4.3	3	1.40
Q99LC5	Electron transfer flavoprotein subunit alpha, mitochondrial	35.0	19.8	5	1.42
Q9DCW4	Electron transfer flavoprotein subunit beta	27.6	31.4	9	1.43
P35550	rRNA 2′-O-methyltransferase fibrillarin	34.3	9.2	2	1.43
Q921G7	Electron transfer flavoprotein-ubiquinone oxidoreductase, mitochondrial	68.0	2.4	2	1.44
P41216	Long-chain-fatty-acid–CoA ligase 1	77.9	19.9	12	1.51
P50247	Adenosylhomocysteinase	47.7	9.5	4	1.59
Q8BW75	Amine oxidase [flavin-containing] B	58.5	2.7	2	1.66
Q8CI51	PDZ and LIM domain protein 5	18.0	35.4	6	1.67
Q91Z83	Myosin-7	222.7	36.5	81	1.73
P19157	Glutathione S-transferase P 1	23.6	12.9	2	1.78
P16125	L-lactate dehydrogenase B chain	36.5	31.1	12	2.07
Q5SX40	Myosin-1	223.2	59.9	148	2.15
P54071	Isocitrate dehydrogenase [NADP], mitochondrial	50.9	30.7	13	2.25
Q8R0Y6	10-formyltetrahydrofolate dehydrogenase	98.6	0.9	1	2.57
P51667	Myosin regulatory light chain 2, ventricular/cardiac muscle isoform	18.9	22.9	3	2.67
O55143	Sarcoplasmic/endoplasmic reticulum calcium ATPase 2	114.8	17.5	19	2.73
P09542	Myosin light chain 3	22.4	45.1	8	3.28
Q810W6	C-X-C chemokine receptor type 1	40.0	1.71	1	3.78

**Table 5:** The identified proteins that exhibit change in abundance in *mdx* diaphragm in comparison to *mdx* extraocular muscle.

Accession	Description	MW [kDa]	ΣCoverage	Σ# Peptides	Fold Change
P08121	Collagen alpha-1(III) chain	138.9	0.8	1	−3.64
Q5SX39	Myosin-4	222.7	57.9	133	−3.46
O08638	Myosin-11	226.9	4.6	8	−3.36
P32848	Parvalbumin alpha	11.9	74.6	15	−2.86
P13541	Myosin-3	223.7	31.7	66	−2.73
Q8R429	Sarcoplasmic/endoplasmic reticulum calcium ATPase 1	109.4	31.7	31	−2.03
Q01149	Collagen alpha-2(I) chain	129.5	4.7	6	−1.99
P11087	Collagen alpha-1(I) chain	137.9	4.9	6	−1.88
O09165	Calsequestrin-1	45.6	23.8	10	−1.78
Q91V92	ATP-citrate synthase	119.7	3.2	3	−1.75
P21550	Beta-enolase	47.0	28.6	14	−1.72
Q9Z1E4	Glycogen [starch] synthase, muscle	83.9	7.6	5	−1.63
Q7TQ48	Sarcalumenin	99.1	27.8	18	−1.63
P13707	Glycerol-3-phosphate dehydrogenase [NAD+], cytoplasmic	37.5	15.5	5	−1.62
P05202	Aspartate aminotransferase, mitochondrial	47.4	22.8	10	−1.62
P21107	Tropomyosin alpha-3 chain	32.8	49.3	21	−1.62
P22599	Alpha-1-antitrypsin 1–2	45.9	24.5	10	−1.59
P19096	Fatty acid synthase	272.3	3.2	7	−1.53
Q00898	Alpha-1-antitrypsin 1–5	45.9	22.3	10	−1.52
Q5EBG6	Heat shock protein beta-6	17.5	21.0	3	−1.46
P46412	Glutathione peroxidase 3	25.4	15.9	3	−1.44
Q3UV70	[Pyruvate dehydrogenase [acetyl-transferring]]-phosphatase 1, mitochondrial	61.1	1.9	1	−1.42
Q6P8J7	Creatine kinase S-type, mitochondrial	47.4	29.1	13	−1.42
P07759	Serine protease inhibitor A3K	46.8	17.5	6	−1.42
P05064	Fructose-bisphosphate aldolase A	39.3	44.5	18	−1.41
Q8CI43	Myosin light chain 6B	22.7	34.3	7	−1.37
Q9WUB3	Glycogen phosphorylase, muscle form	97.2	34.6	30	−1.32
Q9CRB8	Mitochondrial fission process protein 1	18.3	14.5	2	−1.29
Q61147	Ceruloplasmin	121.1	1.5	1	−1.28
Q9CR62	Mitochondrial 2-oxoglutarate/malate carrier protein	34.1	12.4	4	−1.28
Q8BH59	Calcium-binding mitochondrial carrier protein Aralar1	74.5	31.2	15	−1.25
Q924D0	Reticulon-4-interacting protein 1, mitochondrial	43.3	3.3	1	−1.25
P11499	Heat shock protein HSP 90-beta	83.3	24.6	17	1.25
P09103	Protein disulfide-isomerase	57.1	12.8	7	1.26
Q60932	Voltage-dependent anion-selective channel protein 1	32.3	41.6	10	1.29
Q9CZX8	40S ribosomal protein S19	16.1	18.6	3	1.30
Q62009	Periostin	93.1	1.7	1	1.33
Q9CXT8	Mitochondrial-processing peptidase subunit beta	54.6	3.7	2	1.34
P14602	Heat shock protein beta-1	23.0	27.3	5	1.35
Q8VDD5	Myosin-9	226.2	6.3	11	1.36
P15864	Histone H1.2	21.3	33.0	9	1.40
P17742	Peptidyl-prolyl cis-trans isomerase A	18.0	35.4	6	1.43
P14148	60S ribosomal protein L7	31.4	12.2	3	1.44
O09161	Calsequestrin-2	48.2	12.1	5	1.45
Q5SX40	Myosin-1	223.2	59.9	148	1.46
Q9D1R9	60S ribosomal protein L34	13.3	6.0	1	1.47
P47955	60S acidic ribosomal protein P1	11.5	14.0	1	1.49
Q9D0K2	Succinyl-CoA:3-ketoacid-coenzyme A transferase 1, mitochondrial	56.0	8.3	3	1.50
P10107	Annexin A1	38.7	10.4	3	1.51
P19324	Serpin H1	46.6	10.8	3	1.52
P47915	60S ribosomal protein	17.6	5.0	1	1.52
Q9D8N0	Elongation factor 1-gamma	50.0	7.8	4	1.56
P16045	Galectin-1	14.9	5.9	1	1.63
Q9DB60	Uncharacterized protein C1orf93 homolog	21.7	4.0	1	1.71
P51881	ADP/ATP translocase 2	32.9	38.9	12	1.81
P54071	Isocitrate dehydrogenase [NADP], mitochondrial	50.9	30.7	13	1.82
P16125	L-lactate dehydrogenase B chain	36.5	32.6	11	1.87
P51667	Myosin regulatory light chain 2, ventricular/cardiac muscle isoform	18.9	75.7	15	2.07
P11404	Fatty acid-binding protein, heart	14.8	48.9	7	2.19
P09542	Myosin light chain 3	22.4	45.1	8	2.25
Q810W6	C-X-C chemokine receptor type 1	40.0	1.7	1	3.27
O55143	Sarcoplasmic/endoplasmic reticulum calcium ATPase 2	114.8	17.5	19	3.91

Some proteins [41] were found exclusively in the comparison of the *mdx* DIA with the *mdx* EOM and therefore are more likely to be directly involved in the processes of muscle degeneration-regeneration or to the protection against myonecrosis. Regarding their biological processes classifications, most of them (37.2%) were related to carbohydrate, lipid or protein metabolism. Some were related to the immune system processes: in the dystrophic DIA, 3 proteins (HSP beta-6, glutathione peroxidase 3 and ceruloplasmin) were decreased and 5 proteins (HSP 90-beta, HSP beta-1, peptidyl-prolyl cis-trans isomerase A, elongation factor 1-gamma and galectin-1) were increased.

To select proteins that could be directly involved in dystrophic muscle degeneration we made a further double comparison (*mdx* DIA *vs.* control DIA with *mdx* DIA *vs. mdx* EOM) and found 19 proteins in common. Among these proteins, the majority (11 proteins; protein disulfide isomerase, 40S ribosomal protein S19; peptidyl-prolyl cis-trans isomerase; 60S ribosomal protein L7, L29 and L34; 60S acidic ribosomal protein P1; annexin A1; serpin H1; galectin-1 and ADP/ATP translocase 2) were increased in the *mdx* DIA (which presents muscle degeneration-regeneration in comparison to *mdx* EOM). Fewer (6 proteins; myosin-4, calsequestrin 1, ATP-citrate synthase, glycerol-3-phosphate dehydrogenase, fructose bisphosphate aldolase A, reticulon-4-interacting protein 1) were increased in control DIA and in the *mdx* EOM (which do not show muscle degeneration). Two myosins were increased or decreased depending on the comparison: myosin light chain 3 was 30% decreased in *mdx* DIA *vs.* control DIA and 124% increased in *mdx* DIA *vs. mdx* EOM and myosin light chain 6B was 76% increased in *mdx* DIA *vs.* control DIA and 37% decreased in *mdx* DIA *vs. mdx* EOM.

### Western blot analysis

**Figure 2:**
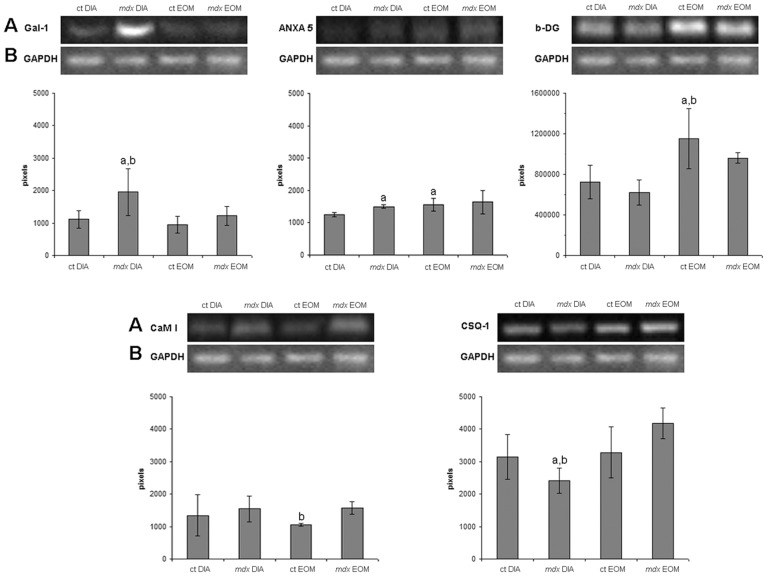
Western blot analysis of some proteins revealed by proteomics. Quantification of galectin-1 (Gal-1), annexin A5 (ANXA5), β-dystroglycan (b-DG), calmodulin I (CaM I) and calsequestrin-1 (CSQ-1) by Western blot analysis in crude extracts of diaphragm (DIA) and extraocular (EO) muscles from control (ct) and dystrophic (*mdx*) mice. In A, Western blot of proteins. In B, the same blot reprobed for GAPDH as a loading control. Graphs represent the level of proteins expressed in pixels. Bars represent standard deviation. ^a^ Significantly different from ct DIA (p≤0.05, ANOVA). ^b^ Significantly different from *mdx* EOM (p≤0.05, ANOVA).

Western blot was performed in order to independently validate the identification and quantification of some proteins in DIA and EOM muscles of control and *mdx* mice (Figure 2). Western blot analysis indicated that galectin-1 levels were significantly higher in *mdx* DIA compared to control DIA (75% increase) and to *mdx* EOM (60% increase; p≤0.05, ANOVA; Figure 2). This is in agreement with the proteomic analysis showing a significant increase of galectin-1 in *mdx* DIA compared with control DIA (fold change of 2.02, Table 2) and with *mdx* EOM (fold change of 1.63, Table 5).

Annexin A5 also presented similar changes as those detected with MudPIT-TMT for most of the comparisons, mainly the increased (20%) levels of this protein in *mdx* DIA *vs.* control DIA (Table 2) and no differences in *mdx* EOM *vs.* control EOM (Figure 2). While Western blot analysis showed lower (20%) levels of annexin A5 in control DIA in relation to control EOM, proteomics showed comparable levels of annexin A5 between these muscles (Figure 2, Table 4).

The proteins related to calcium homeostasis, calmodulin I and calsequestrin-1, also displayed similar expression changes using both Western blot and proteomics analyses. Calmodulin levels were similar among the groups (blot and proteome), with a tendency to be increased in the *mdx* EO compared to control EO (significance only detected by Western blot analysis) (Figure 2), in agreement with our previous work [31]. Calsequestrin-1 was reduced in *mdx* DIA compared with control DIA (23% reduction) and with *mdx* EO (42% reduction; Figure 2) in line with the proteomic finding showing a significant decrease of calsequestrin-1 in *mdx* DIA compared with control DIA (fold change of −1,29, Table 2) and with *mdx* EO (fold change of −1.78, Table 5).

Western blot analysis of β-dystroglycan expression confirmed previous studies [32] demonstrating a persistent expression of this protein in the *mdx* EO concomitant with a significant decrease in the affected muscle (*mdx* DIA; Figure 2C). We also found that dystroglycan (α and β) expression was decreased in *mdx* DIA compared with *mdx* EO (−1.12) and control EO (−1.11) and similar results were described in a recent proteomic study [33].

## Discussion

### Shotgun proteomic analysis

By using the shotgun proteomic analysis, we have here identified a total of 857 proteins in the DIA and EOM from control and dystrophic *mdx* mice at 2 months of age. Out of the 857 proteins, 42 had differential abundance of peptide ions in the DIA of the *mdx* mice. Previous studies using the DIGE proteomic analysis detected 2398 2D spots of which 19 [7] or 35 [8] proteins showed a differential abundance of spots in *mdx* DIA compared to control DIA. By using the 2D gel-based proteomic technique, one protein made from one gene may exhibit an average of 10–15 different spots [34] due to pos-translational modifications and protein degradation [35], [36]. Additionally, spots could appear in the gels due to disulfide bridges because the current DIGE protocol does not require alkylation during the isoelectric focusing step [34]. Therefore, the fact that multiple spots correspond to one protein may explain the difference between the number of spots detected by DIGE and the number of proteins detected in the present study. Nevertheless, the present study demonstrates that the shotgun methodology allowed the identification and quantification of most proteins present in a small amount of muscle (100 µg) and in a short period of time (one run in the mass spectrometer).

In the dystrophic DIA, we observed that some proteins already displayed abnormal levels at this early stage of the disease (2 months of age). Some of the changed proteins detected in the DIA had not been described before in other proteomic studies, such as galectin-1, annexin, serpin H1 and periostin. We also found proteins that had been described by other proteomic techniques. For instance, by using DIGE analysis, malate dehydrogenase, myosin light chain 3, myosin light chain 6B, myosin-4, myosin-9 and vimentin were found to be altered in the *mdx* diaphragm at 9 weeks of age [7], [8]; phosphoglucomutase-1 and phosphoglycerate mutase 2 were changed in the 4–7 week old *mdx* gastrocnemius [12] and 2,4-dienoyl-CoA reductase mitochondrial, myosin light chain 3 and peroxiredoxin were affected in the 9 months of age *mdx* heart [10]; troponin T slow skeletal muscle and four and a half LIM domains protein 1 expression was also affected in *mdx* muscles when comparing the proteomic profile of dystroglycan-interacting proteins [33]. Therefore, the shotgun technique proves to be effective in demonstrating new altered proteins as well as proteins already described by the seminal DIGE studies [7]–[10], [12], [13], [37]. Furthermore, given that the analysis of different samples is performed at one run in the mass spectrometer, we were able to compare the proteomic profile of two different muscles: the affected DIA and the non-affected EOM.

The MudPIT technique has never been used before to investigate the molecular aspects of dystrophin absence in the *mdx* mice. In the present study, we demonstrate that this technique contributes to new insights to the pathophysiology of dystrophy. However, it is important to note that, as with any other technique, the shotgun proteomics has its weaknesses. For improved proteome coverage, optimization of fractionation processes and enrichment of purified organelles could allow a more comprehensive view of the molecular aspects of the disease [20], [38], [39]. For instance, in a recent study of *mdx* limb muscles, immunoprecipitation coupled with shotgun proteomics allowed proteomic analyzes of the dystrophin-associated protein complex *per se* and identification of new dystroglycan-associated proteins [33]. In addition, the combination of different proteomic approaches could lead to a more complete coverage of the proteomic profile [38].

### Proteins related to protection against myonecrosis

The comparison of EOM with DIA muscle revealed altered expression of cytoskeletal proteins (myosin and troponin) and of extracellular matrix components, such as collagen. Furthermore, proteins related to calcium homeostasis and ion channels also exhibited different expression levels. The differential levels of calequestrin1, SERCA1 and SERCA2 observed here, possibly related to fiber type, suggest a better calcium homeostasis, and consequent protection against myonecrosis, in the EOM compared with DIA. This finding is in agreement with previous observations in the *mdx* EOM using Western blotting analysis [31]. Other *mdx* spared muscles, such as the intrinsic laryngeals, also show higher levels of SERCA1 in comparison to normal ILM muscles [40]. Moreover, we observed that the calcium buffering proteins sarcalumenin and calsequestrin 1 [41], [42] were increased in EOM compared to DIA, even in the control group. Therefore, the present results support previous observations (with Western blotting, ELISA and immunocytochemistry techniques) that constitutional properties of the EOM compensate for the lack of dystrophin, allowing a better response against myonecrosis [11], [43].

### Proteins related to degeneration and regeneration

#### Oxidative stress and fibrosis

Proteins involved in the oxidative stress response (HSP 75 kDa, 78 kDa glucose-related protein, serpin H1 or HSP 47 and peroxiredoxin-2) were increased in the *mdx* DIA compared to control DIA. While peroxiredoxins are antioxidant enzymes that control peroxide levels induced by cytokines [44], the HSPs have chaperone functions [45]. The higher levels of these proteins may reflect an attempt of the dystrophic DIA to control oxidative stress at this stage of the disease [46], [47]. However, HSP 47 is also related to increased collagen production and fibrosis [48], which will be morphologically prominent in the *mdx* DIA but only at later stages of the disease [28], as demonstrated by proteomics [13]. Interestingly, several proteins related to fibrosis that were demonstrated to be increased in the old *mdx* DIA muscle compared with younger *mdx* DIA, such as collagen α1 (VI) chain, minecan and actinin-α2 [13] had no differential abundance of peptide ions at the age studied here (comparing *mdx* DIA×control DIA in the present study). This suggests that time-related changes of the proteomic profile occur and this may be of relevance for future studies of drug therapy for DMD.

#### Inflammation, apoptosis and regeneration

We detected an increased level of the annexins A1 and A5 in *mdx* DIA. The annexins bind to negative charged phospholipids in a calcium-dependent manner and participate in many physiological processes, such as cell shape changes, transport and organization of vesicles, exocytosis and endocytosis [49]. Annexin A1 prevents muscle degeneration due to sarcolemma resealing repair [50], [51]. Extracellular annexins act in fibrinolysis, coagulation and apoptosis [52], [53]. The annexins A1 and A2 are overexpressed in different forms of muscular dystrophies, possibly related to their anti-inflammatory activity [54]. Annexin A1 is a glucocorticoid-inducible protein [55], [56] able to mimic the anti-inflammatory effects of glucocorticoids in several experimental models of inflammation both *in vivo* and *in vitro* [57]. Besides participating in apoptotic processes, annexin A5 presents anti-inflammatory properties by inhibiting phospholipase A2 and phosphadylserine-catalyzed inflammation [58], [59]. Therefore, the increased level of annexins early in the *mdx* DIA may suggest a potential ability of the dystrophic DIA to modulate inflammation.

Reticulon-4-interacting protein 1 is a mitochondrial protein that reduces the anti-apoptotic activity of Bcl-2 and Bcl-XL [60], [61] and was decreased in the *mdx* DIA compared to control DIA. This finding indicates that apoptosis may be involved in *mdx* pathology [62]–[66], at least during the early phase, although apoptotic fibers have not been consistently detected in the *mdx*, at later stages [67], [68]. Galectin-1 was also overexpressed in *mdx* DIA. This protein is produced by myoblasts and other cell types [69], [70], and participates in muscle regeneration [71]–[76], and may be involved in the regenerative ability of the dystrophic DIA during this period, since at later stages fibrosis is a hallmark of diaphragm dystrophy. Galectin-1 also has a protective effect on skeletal muscle by reducing inflammation [77].

### Conclusions

In the present study we demonstrated that the shotgun proteomics approach adds to the former proteomic techniques [7]–[10], [12], [13], [37] as a suitable alternative to track possible changes in the levels of proteins in dystrophic muscles, during the early phase of the disease. We would like to highlight some advantages of the technique that include the small amount of sample required, the relatively short time to accomplish the analysis and the possibility to perform qualitative and quantitative comparisons between distinct muscles and experimental groups.

The current proteomics study of the dystrophic DIA, in the phase prior to more advanced disease [13], [26], demonstrates an increase in proteins involved in muscle regeneration (galectin-1 [76], [77]), calcium handling (calsequestrin 1 [31]), inflammation (annexin A1 [50], [51], [54]) and fibrosis (HSP 47 [48]), making them valuable candidates for being potential drug targets and exploratory biomarkers.

## Supporting Information

Table S1 **Proteins identified in extraocular and diaphragm muscles of control and** ***mdx*** **mice in three biological replicates.**
(XLS)
